# Domestic Use Simulation and Secondary Shelf Life Assessment of Industrial *Pesto alla genovese*

**DOI:** 10.3390/foods10081948

**Published:** 2021-08-21

**Authors:** Carola Nicosia, Patrizia Fava, Andrea Pulvirenti, Andrea Antonelli, Fabio Licciardello

**Affiliations:** 1 Department of Life Sciences, University of Modena and Reggio Emilia, 42122 Reggio Emilia, Italy; carola.nicosia@unimore.it (C.N.); patrizia.fava@unimore.it (P.F.); andrea.pulvirenti@unimore.it (A.P.); andrea.antonelli@unimore.it (A.A.); 2 Interdepartmental Research Centre for the Improvement of Agro-Food Biological Resources (BIOGEST-SITEIA), University of Modena and Reggio Emilia, 42122 Reggio Emilia, Italy

**Keywords:** household food waste, stability evaluation, sensory acceptability, period after opening (PAO)

## Abstract

The secondary shelf life (SSL) is defined as the time after package opening during which the food product retains a required level of quality. The SSL, indicated in labels as “best if used within *x* days after opening”, could lead to domestic food waste if not correctly evaluated. In this context, the SSL of two brands of industrial shelf-stable pesto products (with an indicated SSL of 5 days) was studied through a domestic use simulation performed in five households under two scenarios simulating real opening and storage conditions. The quality of pesto after opening was assessed through microbiological and sensory analyses, determination of instrumental colour parameters, pH and volatiles profiling. For both pesto sauces tested, a SSL ≥ 20 days was proven. Irrespective of the intensity of use (scenarios 1 and 2), the pesto was microbiologically stable: the maximum count for total aerobic mesophilic bacteria (TMB) observed during 20 days of storage was 9.64 ± 1.7 × 10^2^ CFU/g, starting from a commercially stable product. Colour parameters L* and ΔE did not change significantly during storage (*p* > 0.05), while the a* and BI values significantly changed (*p* < 0.05) during the first 5 days, and then stabilized during the rest of the household storage. Nevertheless, the slight colour modifications were not perceived by the sensory panel. Moreover, sensory assessors were not able to discern pesto samples stored for up to 20 days after first opening, from a just-opened reference sample, proving that the sensory appreciation of pesto was not influenced by the time after opening. The results of this study suggest the possibility to significantly extend or even omit the SSL indications for industrial pesto sauces. The objective assessment of SSL could have impressive practical outcomes both for the industry and the end user. The elongation of the SSL on the food label might increase food sustainability, thanks to the potential reduction of food wastes, thus giving added value to the commercial products. In addition, the end user could benefit the increase of the useful period for the food consumption after first opening, with significant domestic food waste reduction, reduced household stock turnover and consequent cost savings.

## 1. Introduction

In the context of sustainability improvement in the food sector, various measures have been proposed, especially related to the mitigation of environmental impacts of processes and materials, the optimization of distribution and logistics, and the minimization of food losses and wastes (FLW) along the food chain [1]. It is widely accepted in the scientific community that FLW are responsible for a high fraction of global environmental impacts [2,3]. FLW represent an economic, social, and environmental issue, and for this reason, the EU has targeted the halving of food wastes by 2030, according to Sustainable Development Goals (SDGs) [4]. Specifically, SDG 12.3 aims to “by 2030, halve per capita global food waste at the retail and consumer levels and reduce food losses along production and supply chains, including post-harvest losses”. The most recent estimates report that 17% of overall food production is wasted [5]. Most of this waste is produced in the downstream of the chain, especially at the household level: available data range from 33–38% [6], through 45% [7] to 61% of total FLW [5]. Recent evidence [5] has shown that consumer food waste has been significantly underestimated and that figures related to food waste at consumer and food service level (also referred to as “avoidable food wastes”) are more than twice as much as previously estimated [6].

It should be highlighted that domestic food waste: (i) cannot find alternative uses; (ii) represents the highest fraction of total FLW and (iii) is responsible for the highest environmental impact, since wasted products represent the highest level of resource consumption and emissions. Indeed, the resource consumption necessary for food production is in vain when food is lost or wasted and misses its goal of human consumption [8,9]. Given the need for FLW prevention and based on the awareness of the importance of domestic food waste [10], it is urgent to adopt effective mitigation measures.

Household food waste reduction could be achieved by the downsizing of packages. Smaller packages, indeed, reduce the probability of not being able to consume the product within an appropriate time. However, this approach brings about a higher consumption of packaging materials per food unit. In many cases, it is the overly short secondary shelf life (SSL), i.e., the timespan from the first opening to unacceptability, that turns food products into wastes. This timespan, also referred to as “period after opening” (PAO) is mandatory for some cosmetics [11]; for foods, it is usually communicated to consumers through the package label in terms of instructions for use after the first opening with sentences such as “after opening, store refrigerated and consume within *x* days”, where *x* ranges from 24 h to a few days, depending on the product category and, within the same product category, depending on the producer. Such indications, however, does not have any scientific support and may even mislead consumers, thus contributing to foods which are still perfectly suitable for consumption being discarded.

*Pesto alla genovese* is a traditional Italian sauce made from basil, olive oil, grated hard cheese, pine seeds, salt, and garlic commonly used as a dressing for pasta. It is widely available as a shelf-stable product in glass jars, with a shelf life of 2–3 years; however, its stability after opening has never been assessed. Therefore, the aim of this work was to estimate the secondary shelf life of Italian *pesto alla genovese* by simulating two levels of domestic use and storage in five different house environments and through monitoring microbiological, sensory and chemical quality descriptors. The study took into consideration two popular commercial brands and compared the experimental results with the respective label-indicated secondary shelf lives.

## 2. Materials and Methods

### 2.1. Products

Two shelf-stable commercial pesto sauces from different manufacturers, identified here as P1 and P2, were used in this study, both packaged in a 190-g glass jar with a metal cap and having a similar composition. The composition listed on the label of P1 was: sunflower oil, fresh basil 30%, cashews, Parmigiano Reggiano PDO 5%, corn fibre, whey powder, salt, milk protein, extra virgin olive oil, sugar, basil extract, natural flavours, lactic acid, garlic. P2 had the following composition: Genoese basil PDO 35%, sunflower oil, cashews, extra virgin olive oil 10%, Grana Padano PDO 6%, Pecorino Romano PDO 4%, salt, pine nuts, lactic acid, garlic and ascorbic acid.

Pictures of P1 and P2 are available in Figure S2, in the Supplementary Material. The average nutritional values of the two commercial pesto sauces are shown in Table 1. Both products had a residual primary (commercial) shelf life of at least one year when the experiments started. The SSL reported on the labels of both P1 and P2 was 5 days from opening, under refrigerated storage.

### 2.2. Simulation of Domestic Use and Sampling Plan

The SSL assessment was performed using a deterministic approach, as suggested by Nicoli and Calligaris [12], which consists in storing the opened food product under the expected environmental conditions (i.e., at home in the case of shelf-stable pesto) and the worst-case scenario. The pesto samples were divided into five lots, each referred to a different domestic environment (coded from A to E). Pesto jars were stored at ambient temperature until use, according to protocols described hereafter. Among the possible way to simulate the worst-case found in literature, repeating the container opening and closure during the household storage was the selected approach for this study [12]. Two levels of use (referred to as “scenarios”, based on the number of openings and duration of each opening) were tested in the five home environments mentioned above, to simulate a real utilization by the consumer (Table 2), while the analytical determinations were carried out at the laboratories of the Department of Life Sciences of the University of Modena and Reggio Emilia. This is an innovative feature that distinguishes this study from most of the research in the field of SSL [13,14,15].

Scenario 1 (S1) consisted of a single opening for each jar, corresponding to the beginning of SSL, hence referred to as time zero (t_0_). Two tablespoons of pesto were removed from each jar, which remained uncapped for 2 min, then the jars were closed and stored in the domestic refrigerator, where they were kept for 20 days.

Scenario 2 (S2) consisted of three openings of each jar at 2-days intervals. At each opening, two tablespoons of pesto were removed from each jar, which remained uncapped for 3 min, then the jars were closed and kept at ambient temperature for 30 min before placing in the domestic refrigerator, where they were stored for 20 days.

Dataloggers (mini-TH, XS Instruments, Carpi, Italy) were used to monitor conditions of the five domestic refrigerators where samples were stored, by recording temperature and humidity at 1-h intervals up to 168 h (one week).

The opening procedure described above for each scenario was performed simultaneously on four pesto jars in each home environment. Following the opening protocols, pesto jars were stored in domestic refrigerators and delivered to the laboratory on day 5, 11, 16, and 20 after the first opening. On the scheduled day, microbiological, sensory, and chemical-physical analyses were performed on the pesto samples from the five domestic environments.

### 2.3. Microbiological Analysis

Microbiological analyses were performed to assess the degree of contamination of pesto samples during refrigerated storage after first opening, through total aerobic mesophilic and *Clostridium* counts.

Ten grams of each sample were diluted with 90 g of sterile physiological solution (0.9% NaCl) in sterile stomacher bags and homogenized in a laboratory Stomacher 400 blender (Seward Limited, Worthing, UK) at high speed for 60 s. Serial dilutions were prepared in sterile physiological solution. The pour plate method was performed for the aerobic mesophilic count, using Plate Count Agar (PCA, Tryptic Glucose Yeast Agar, Biolife, Milan, Italy) and the plates were incubated at 30 °C for 48–72 h. For *Clostridium* counts, the prepared samples underwent thermal shock at 95 °C for 10 min, before pouring in plate with *Clostridium* Agar (Biolife). The plates were incubated anaerobically at 30 °C for 48 h. Anaerobic conditions were obtained using Oxoid AnaeroGen bags (Thermo Fisher Scientific Inc., Waltham, MA, USA) inside anaerobic jars. All experiments were performed in duplicate. The results are expressed as colony-forming units (CFU) per gram of pesto.

### 2.4. Sensory Evaluation

The sensory test was performed on day 5, 11, 16, and 20 after the first opening. A panel of 12 judges (six males and six females, aged 23 to 63) was selected among the staff of the Department. The panelists were familiar with sensory evaluation and had previous experience in testing food through the triangular test. The sensory analysis aimed to understand the acceptance of samples during household storage. Triangular tests were performed on pesto samples from each of the five domestic conditions, presented together with a reference sample, which belonged to the same production batch (same formulation, processing, and storage conditions) but freshly opened. The reference jar, even if not opened, was also stored in the refrigerator before the sensory evaluation. Each assessor was asked to evaluate five triplets of randomly coded samples. Each set contained two identical samples and one different sample, which was randomly selected between the stored pesto (from home environment A, B, C, D or E) and the control one. The objective of the sensory evaluation was to assess: (i) whether judges were able to discriminate the stored samples from the control (just opened) one, and (ii) which was the degree of acceptance of samples throughout the time after the first opening.

Randomization was applied in the presentation order for all the panelists. All the samples were served at the same temperature to avoid the “stimulus error” due to the preparation of the samples. The participants were provided with unsalted crackers and water to be used during the tasting session. The judges were asked to select the different sample within each triplet, and to express its overall acceptability on a scale from 0 (extremely dislike) to 10 (extremely like). Figure S1, found in the Supplementary Material, shows the pesto samples prepared for the sensory analysis.

A significance level of 5% was chosen (α = 0.05), which means accepting a 5% risk of finding a difference when there is none. Using an α value of 0.05 and a 12-member panel, the minimum number of correct answers to reject the assumption of no difference is 8 [16].

### 2.5. Measurement of Colour Parameters

The determination of colour was carried out at day 5, 11, 16, and 20 after first opening. Colour was measured using a chroma-meter CR-400/410 (Minolta Camera, Co., Ltd., Osaka, Japan) with a window diameter of 8 mm, equipped with a standard illuminant D65, and 10° observer angle. The values of the CIEL*a*b* parameters L* (lightness), a* (redness/greenness), and b* (yellowness/blueness) were recorded.

The chroma-meter was calibrated using a white ceramic tile (Y: 93.7, x: 0.3134, y: 0.3195) before the determination. For the colour assessment, a standardized amount of pesto (8 g) was weighed, and the colour was measured on three different points of each sample and reported as mean value ± standard deviation.

The total colour difference (ΔE) and the browning index (BI) of samples were calculated as follows [17,18,19]:

(1) $$\Delta E=\sqrt{{\left(L^{*}{}_{0}{-L}^{*}{}_{t}\right)}^{2}+{\left(a^{*}{}_{0}{-a}^{*}{}_{t}\right)}^{2}+{\left(b^{*}{}_{0}{-b}^{*}{}_{t}\right)}^{2}}$$

(2) $$\mathrm{BI}=\frac{100\times \left[\left[\frac{a_{t}^{*}+\left(1{.75\times L}_{t}^{*}\right)}{\left(5{.645\times L}_{t}^{*}\right){+a}_{0}^{*}-\left(3{.012\times b}_{t}^{*}\right)}\right]-0.31\right]}{0.17}$$

### 2.6. Determination of a_w_ and pH

Water activity (a_w_) and pH were determined on the products at the time of first opening (t_0_) and pH was also monitored at day 5, 11, 16, and 20 after first opening. The a_w_ of pesto samples was measured using the dew point AquaLab 4TE water activity meter (Meter Group, Inc., Pullman, WA, USA). A standardized amount of sample (4 g) was weighed and brought to ambient temperature before the a_w_ determination.

To measure the pH, the samples were homogenized with the physiological solution and the pH was measured using a CyberScan pH 310 (Thermo Fisher Scientific Inc., Waltham, MA, USA) after calibration with buffer solutions at pH 7.00 and 4.00 (Sigma-Aldrich, Merck KGaA, Darmstadt, Germany).

The a_w_ and pH were additionally measured on ten commercially available shelf-stable pesto sauces, to have an overview of these parameters within the product category. All a_w_ and pH determinations were conducted in triplicate.

### 2.7. HS-SPME-GC/MS Volatiles Profiling

Volatile organic compounds (VOCs) of pesto sauce were analyzed by headspace solid-phase micro extraction (HS-SPME) followed by gas-chromatography/mass spectrometry (GC-MS) analysis just after opening and following refrigerated storage for 20 d from the first opening, in order to assess possible changes in the volatile fraction.

Two grams of just-opened P1 and P2 pesto and of each of the five samples (one from each household) stored for 20 d after opening, were weighted into 25-mL screw-cap glass vials provided with Mininert^©^ valves (Merck KGaA, Darmstadt, Germany). Vials were conditioned at 50 °C for 15 min in a thermoblock (Falc Instruments, Treviglio, Italy), then a divinylbenzene/carboxen/polydimethylsiloxane (DVB/CAR/PDMS) SPME fibre was exposed in the headspace for 30 min at the same temperature for the extraction of volatile compounds. Chromatographic separation of analytes was carried out by an Agilent GC-MSD (7890A/5975C, Agilent Technologies Inc., Santa Clara, CA, USA) provided with a Stabilwax-DA (0.25 mm i.d. × 30 m × 0.25 μm) capillary column (Restek, Milan, Italy). After extraction, fibers were desorbed for 3 min into the GC injector port set in splitless mode at 240 °C. The GC carrier gas used was helium at 1 mL/min and the detector temperature was set at 240 °C. GC oven temperature program was: start at 50 °C for 3 min, 5 °C/min until 160 °C, hold at 160 °C for 2 min, 20 °C/min until 240 °C, hold at 240 °C for 2 min. Peak identification was carried out by comparison with system libraries (Wiley, NIST). The analyses were performed in duplicate.

### 2.8. Statistical Analysis

Statistical analysis was performed by one-way analysis of variance (ANOVA) followed by Tukey’s multiple range test (*p* < 0.05) using the SPSS statistical software (SPSS 20 for Windows, SPSS Inc., IBM, New York, NY, USA). The results were expressed as mean ± standard deviation (SD).

## 3. Results and Discussion

### 3.1. Simulation of Domestic Use

**Figure 1:**
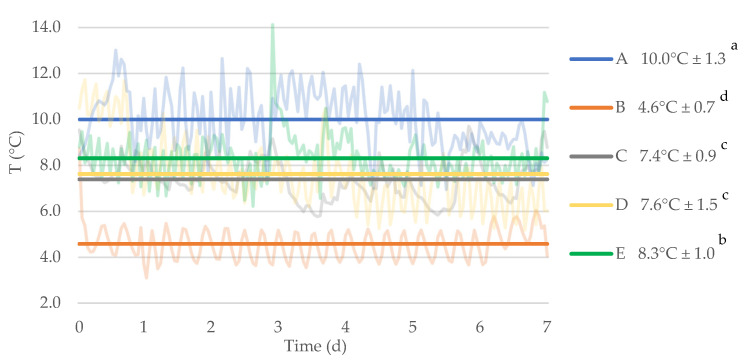
Temperatures (°C) recorded in the five domestic refrigerators (A to E) during one week. Values on the right are given as mean ± SD (*n* = 168). Faded lines represent the temperature profiles (data recorded at 1-h intervals), while the bold horizontal lines indicate the mean values measured for each household. Different superscript letters in the legend indicate significant differences (*p* < 0.05) among mean values.

Jars of *pesto alla genovese* were opened, used, and stored in five households (labelled from A to E), as described in the Materials and Methods section. For each home environment, the refrigerator temperatures were monitored for one week using dataloggers. The temperature profiles during one week and the mean temperatures of the five households are reported in Figure 1.

The values measured in the refrigerators of the five households ranged between 3.1 °C (in household B) and 14.1 °C (in household E). As regards mean values calculated across the monitoring period, the minimum recorded temperature was 4.6 °C ± 0.7 (household B) while the highest mean value was 10.0 °C ± 1.3 (household A). Interestingly, significant differences (*p* < 0.05) were observed among the mean temperatures of the five domestic refrigerators. Some of the domestic refrigerators, such as the ones of households D and A, showed a higher fluctuation of temperatures (hence higher standard deviations) than others, whose temperatures oscillated in a tighter range, as for domestic refrigerators B and C. Overall, the households used for this study covered a wide range of temperatures and could be well representative of different possible conditions of domestic storage of food products after opening.

### 3.2. Microbiological Analyses

For the sake of conciseness, results of microbiological analyses have been condensed in Table 3, which shows the highest counts for total aerobic mesophilic bacteria (TMB) and Clostridia, expressed as CFU/g, observed in the five home environments on each sampling day. The microbial counts in the table are shown based on the scenario, type of pesto (conditions S1P1, S2P1, S1P2, S2P2), and category of microorganisms (total aerobic mesophilic bacteria and Clostridia).

Since the shelf-stability of commercial pesto sauce results from a sterilizing heat process able to destroy all vegetative forms of microorganisms, the product remains commercially sterile until the first opening. For this reason, the degree of contamination of the samples depends on the contamination of the domestic environments in which the opening took place, while the storage temperature could influence the growth rate of microorganisms.

As it can be inferred, the maximum TMB count found within S1P1 was 964 CFU/g for sample C at 5 days after opening. For the same pesto and scenario (S1P1), on the following days, TMB counts did not exceed 32 CFU/g. This behaviour, in which the growth of microorganisms was not increasing throughout the storage time, was noticed more than once in this study. Probably, it could be due to the utilization of a different jar of pesto for each day of sampling, although they were opened at the same time, and treated and stored in the same conditions. In the second scenario of P1, the maximum TMB count (964 CFU/g) was recorded on the 11th day of household storage on sample E. It is worth recalling that each sample was analysed only once, and that at each sampling time a different jar was taken. Even if the approach does not allow to follow growth kinetics, the general trend of counts observed in both scenarios allows to state that *pesto alla genovese*, during the period after opening, has high microbial stability and that the contamination by environmental microbes occurring upon opening and use, is not followed by exponential growth at any storage temperature.

In the first scenario of P2, very low TMB counts were observed, with the highest count of 104 CFU/g for sample E at 5 d after the first opening. As for P1, also P2 showed lower TMB counts during storage, thus confirming the high stability of this product category. In the second scenario of P2, higher TMB counts were observed compared to S1, 793 CFU/g being the maximum values recorded for sample E after 11 d of household storage. Despite the higher degree of use in S2, consisting of 3 openings with product withdrawal, TMB counts remained at very low levels for any of the household conditions. This suggests that neither the household environment contamination nor the storage temperature, even in the worst scenario, can significantly affect the microbiological quality of *pesto alla genovese.*

The highest TMB count observed amongst all samples was 964 CFU/g, which is more than three orders of magnitude lower than the maximum acceptable microbial growth in food of 10^6^ [20] and even lower than the threshold of 10^7^ CFU/g, which indicates spoiled foods [21]. For the sake of completeness, also the maximum recorded counts were subjected to comparison by ANOVA, which highlighted significant differences among treatments, without any clear indication on the effect of the scenario on the microbial load. Significant changes were not found on the TMB counts amongst the scenarios and the type of pesto used (*p* = 0.8). On the other hand, despite significant changes during storage time were found (*p* < 0.05), it should be noted that in most of the cases the microbial load observed 20 d after opening was not significantly different from the one detected at day 5.

The *Clostridium* growth in S1P1 was observed only at day 20 of storage at a very small extent (5 CFU/g) in three samples out of five (A, B, C). In S2 it was slightly higher than the one observed in S1, with a maximum of 55 CFU/g after 16 d in sample D.

In both Pesto sauces P1 and P2, higher *Clostridium* counts were observed in the second scenario, possibly because of the higher number of openings and the longer exposure to the environment (jar kept uncapped), which could have increased the probability of contamination. Interestingly, in P1 it was noticed a higher *Clostridium* growth than the one observed on P2, in which only one sample (A at t_16_) showed Clostridia. The different pH and a_w_ values of the pesto sauces, however, do not influence the degree of contamination by *Clostridium*, but the germination of spores that are already present. The different degree of *Clostridium* contamination between the two pesto sauces could be due to the household environment. To corroborate this hypothesis, *Clostridium* counts for S2P1 were only observed in household D, suggesting that this environment is the source of contamination during the openings of the jars.

The selective parameters (pH and a_w_) of both pesto sauces are able to prevent the germination of *Clostridium* spores, and the *Clostridium* counts observed are a consequence of the germination induced by the thermal shock. The limiting pH for germination of *Cl. botulinum* spores is 4.8–5.0 [22] hence, the very low pH of P2 (4.18) avoids the germination of any *Clostridium* spore. On the other hand, the pH of P1 (5.64) alone could allow spore germination, but this event is unlikely thanks to the very low a_w_ value (0.9046), taking into account that in optimum conditions (30–40 °C and pH 7.0) the a_w_ limiting *Clostridium* spores’ germination is 0.94–0.97 and by decreasing the pH of the medium, the limiting a_w_ increases [22].

### 3.3. Sensory Evaluation

Sensory evaluation was included in this study since sensory perception throughout the period after opening is complementary to hygienic quality assessment: indeed, the end of SSL could depend on microbiological and/or sensory thresholds, as well as primary shelf life can be determined as the lowest value between microbial acceptability limit and sensory acceptability limit [23].

The judges were asked to indicate the different sample within each triplet, anyway, the discrimination of the aged samples from the reference did not necessarily indicate non-acceptability; for this reason, panelists were asked to rate the acceptability of the sample recognized as different.

For P1, it was found that sample E was perceived as different from the reference (just-opened) sample after 11 d from opening, with eight correct answers out of 12. Nevertheless, the evaluations of the judges were still positive: 100% of the eight panelists graded the overall acceptability higher or equal to 6, with mean overall acceptability of 7.6. Moreover, 37.5% of the judges described the sample as: “creamy and with a delicate flavour”, “softer flavour”, “very fragrant”. The level of acceptability within every single triangular test was compared with the acceptability value of the just-opened sample taken as a reference. At the other sampling times, S1P1 samples from the five households were not discriminated from the reference pesto. Concerning the second scenario (S2P1), only one sample (E, 20 d after first opening) was significantly perceived as different. As for S1P1, the sample recognized as different scored in all cases ≥6 for overall acceptability, with a mean value of 7.8. Two judges gave a grade of 9 and described the sample as and “very tasty” “having more delicate flavour”. Based on the panelists assessment, the only two samples of P1 which were recognized as different were not perceived as worse than the control.

In the first scenario of P2, 5 d after opening, three samples (A, D, and E) were discriminated from the reference pesto. However, assessors who were able to recognize the difference indicated the aged samples as “less acid”, “less bitter”, “less sour”, and “more fragrant” than the reference pesto. Similarly, 11 d after opening, sample B was recognized as different from the reference, but its overall acceptability (6.7) was even higher than that of the reference sample (5.6). At 16 and 20 d after opening, none of the samples resulted significantly different from the reference product. In S2P2 the only differences correctly recognized by the panelists (8–9 corrected answers out of 12) were after 11 (sample B) and 20 d (samples C and D) from opening. Anyway, the first sample received overall acceptability of 5.2, only 0.5 points less than the reference (5.7). Samples C and D were graded 6.3 and 7.2, respectively, compared to 6.2 of the reference.

As for P1, the analysis of the judges’ evaluations for P2 showed that, in most cases, aged samples could not be discriminated from the reference (just-opened) sample and that differences correctly assigned did not indicate a decrease of sensory quality of pesto during domestic storage in none of the tested scenarios.

In general, the sensory analysis proved that assessors were generally not able to discriminate just-opened pesto from the product which had been opened up to 20 days before, irrespective of the degree of use. For both pesto brands, some of the judges found it difficult to assess differences amongst the samples, even in the worst scenario (S2) and after 20 d from first opening. Figure S2 (Supplementary Material) shows pictures of the two commercial pesto sauces stored for 20 days after first opening.

No relation between the degree of microbial contamination and sensory perception of pesto samples was observed: indeed, on one hand, some samples with null growth or very low counts were correctly discriminated (8 corrected answers out of 12) while, on the other hand, the samples with the highest microbial contamination were not discriminated from the reference pesto by the sensory analysis.

### 3.4. Colour Measurement

Colour modifications during storage are mainly due to degradative processes such as phenols oxidation, chlorophylls degradation, and nonenzymatic browning [20]. The instrumental colour parameters L* and a* and the derived parameters ΔE, and BI were assessed to evaluate colour modifications of pesto during 20 d of household storage after first opening. The b* parameter was not considered since several authors suggested using a* for green fruits and vegetables, because of its correlation to both green intensity and consumer acceptance [24,25].

Significant changes in lightness (L*) (Table 4) were found during domestic storage after opening only in a few samples of P1 and, in particular, for sample D in S1 and samples D and E in S2 (*p* < 0.05); however, this change was not perceived by the judges during the sensory evaluation. No significant difference was found between the two scenarios (*p* = 0.08). Similarly, for P2 lightness did not differ significantly between the two scenarios (*p* = 0.44) and significant differences of L* during storage occurred only for samples A and D in S1 (*p* < 0.05). It should be noted, however, that the L* value after 20 d from opening was not significantly different from the reference (just-opened) sample.

Since no significant difference was observed between scenarios for neither of the two pesto sauces, it can be inferred that the conditions of use did not influence the lightness value during the time after opening. Overall, the lightness value of the two pesto sauces underwent only slight modifications in a few samples, which however were perceived as acceptable by the sensory panel, hence not influencing the suitability for consumption of the products.

The parameter a* represents the red-green axis of the CIEL*a*b* colour system, in which negative values indicate the greenness colour intensity [19]. This value was used as a quality parameter to evaluate the colour changes of fresh pesto [24] and in the shelf life assessment of pesto [20]. Values of a* of P1 and P2 during storage after opening are reported in Table 4.

Significant changes (*p* < 0.05) were found during domestic storage after opening only in sample C for S1P1 and in samples B and D for S2P1. Anyway, by considering altogether the values of the samples from the five domestic environments and of both scenarios, the a* parameter of P1 was not significantly different during household storage (*p* = 0.08). A significant difference during time after opening was observed for S1P2 in samples A, B, C, and D and for S2P2 in samples A, C, and D. In general, changes in a* occurring in P2 highlight a slight decrease (i.e, a slight increase of greenness) during the first 5 days, followed by stabilization. A comparison between a* values from the two scenarios highlighted no significant differences at each sampling time (*p* = 0.46).

Zardetto and Barbanti [20] studied the variation of greenness in fresh pesto, finding that the a* values increased during the primary shelf life. The difference with our findings may be due to the different products since the authors focused on a refrigerated, minimally processed pesto. On the other side, the pesto sauces used in this study had undergone a more intense thermal treatment (allowing shelf-stability) with subsequent degradation of chlorophyll and colour modification: as described by Zeppa and Turon [24], indeed, the thermal treatment causes an increase of the a* value of pesto.

**Figure 2:**
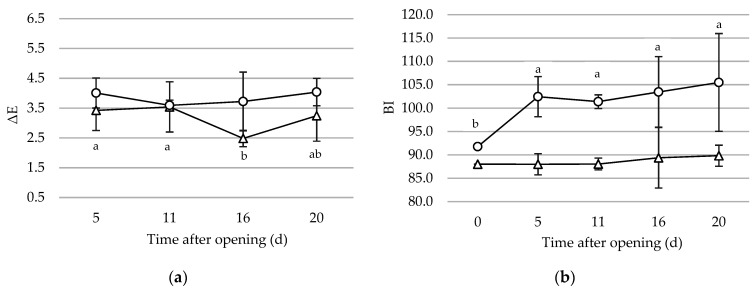
(**a**) Overall color variation (ΔE) and (**b**) Browning Index (BI) for pesto 1 (triangle) and pesto 2 (circle) during household storage after first opening. Each line reports the mean value of samples from the five households and managed under the two scenarios. Different letters within each line indicate significant differences (*p* < 0.05). If letters are not provided, no significant difference was observed.

Often, derived colour indexes have proved to be more useful to assess colour changes compared to primary parameters; for this reason, the overall colour variation (ΔE) and the browning index (BI) were calculated and the trend throughout storage has been reported in Figure 2. For both P1 and P2, the ΔE values did not differ significantly between the two scenarios (*p* = 0.29 and 0.16, respectively), whereby the ΔE values of both scenarios of the same pesto sauce have been condensed and shown in a single broken line, each for one type of pesto. Statistical analysis revealed differences throughout the period after opening only for P1, however, mean values at 5 and 20 d were not significantly different. In general, it can be inferred that ΔE for both pesto sauces remained stable during refrigerated storage after opening.

BI can reveal a possible browning of the product, which in pesto sauce is a consequence of a nonenzymatic process, due to the reaction between proteins and oxidized lipids [20]. In P1, no significant difference of BI values between S1 and S2 was observed (*p* = 0.27), as well as during the period after opening (*p* = 0.32). On the other hand, the BI of P2 was found to change significantly during the first 5 days of storage after opening (*p* < 0.05). Similarly to P1, no significant difference (*p* = 0.13) was observed between the scenarios. The difference in BI between the two types of pesto may be due to their different a_w_ value, which is considerably lower in P1 (0.9046) as compared with P2 (0.9554): indeed, as suggested by Severini et al. [26], nonenzymatic browning is strictly related to a_w_ values.

Overall, results suggest that the primary and derived colour parameters of both shelf-stable pesto brands after opening were not influenced by the level of domestic use. Moreover, colour changes are hardly noticeable throughout the period after opening, and for this reason, they were not perceived by the sensory panel.

### 3.5. Chemical Analysis

The lowering of a_w_ and the reduction of pH are crucial factors for the stabilization of pesto sauce, to achieve microbiological stability and lowering degradative reactions such as browning, lipid oxidation, enzymatic reactions and protein denaturation [26]. The stabilization of pesto sauce is reached both by lowering the pH and/or the a_w_, depending on the producer strategy and on possible taste consequences of the formulation. Low values of a_w_ are generally reached by the addition of humectants such as sugars, salts, polyols and protein derivates [26], while the pH is lowered by formulation with lactic, citric and ascorbic acid, and glucono-δ-lactone.

Along with the two pesto brands object of this study, the pH and a_w_ of 8 other commercial brands of shelf-stable *pesto alla genovese* sauces were determined. Results are provided in Table 5, which show a_w_ ranging from 0.8447 (in P10) to 0.9667 (P9) and pH values ranging from 4.00 (P3) to 5.64 (P1). The a_w_ values of the pesto sauces used in this study are higher than reported in the literature by Fabiano et al. [27] for industrial pesto (0.82), while they agreed with data reported by Severini et al. [26] (ranging from 0.914 to 0.956). As it can be inferred, the pH and a_w_ values of the samples object of the study (P1 and P2) fall in the range of commercial products, except for the pH of P1 which was the highest among commercial brands. Hence, based on stability-related parameters, P1 and P2 well represent the pesto category, and the observed results for these two brands may be extended to the commercial product category of pesto sauces.

**Figure 3:**
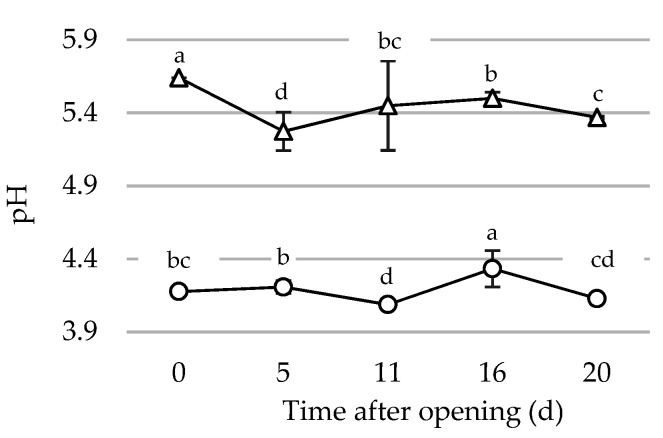
Trend of pH values for Pesto samples (P1: triangle; P2: circle) during household storage after first opening. Each line reports the mean value of samples from the five households and managed under the two scenarios. Different letters within each line indicate significant differences (*p* < 0.05).

The monitoring of pH (Figure 3) did not show significant differences based on the level of domestic use in both pesto sauces (*p* = 0.21 and 0.06 for P1 and P2, respectively), therefore the values from both scenarios were analyzed together. Very small changes, though statistically significant, were shown during the period after first opening (*p* < 0.05) in both pesto brands. In spite of that, the pH of Pesto samples after 20 d from the first opening was very similar to the initial one, thus confirming the intrinsic stability of the products.

### 3.6. Volatile Organic Compounds Profiling

*Pesto alla genovese* is appreciated for its flavour, which results from its specific formulation based on basil, hard cheese, garlic, and nuts. The sensory perception of Pesto might change after opening due to loss of key odorants, and oxidation reactions of volatiles and/or fatty components. The sensory assessment of pesto samples during the period after opening did not reveal significant changes perceived by tasters. A further investigation on the volatile fraction was aimed at confirming the stability of pesto during the time after opening and at determining possible changes which are not detected by sensory assessment. Hence, the volatile profiles of P1 and P2 sampled at the time of first opening were compared with those of samples stored refrigerated for 20 d after first opening, managed under scenario S2, which implied a more intensive level of use (worst case).

**Figure 4:**
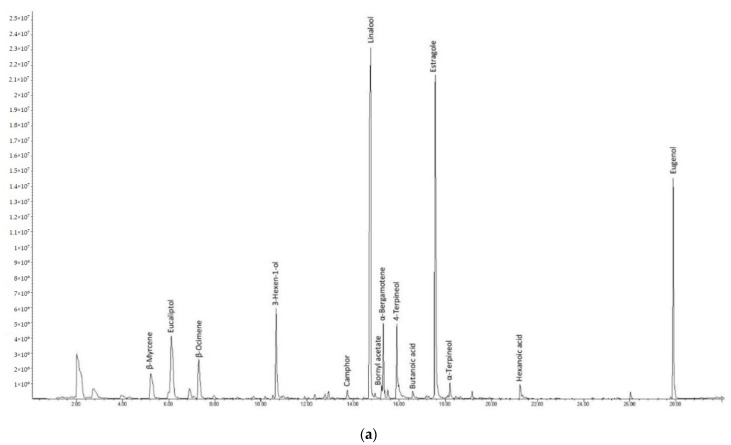

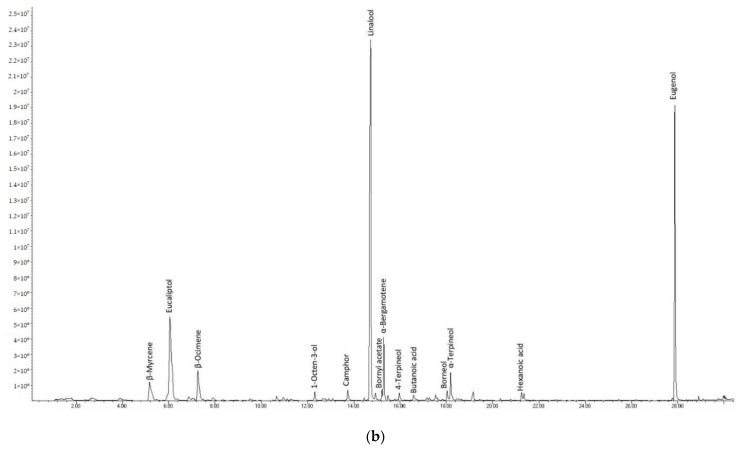
Typical chromatogram of the volatile fraction of pesto (**a**) P1 and (**b**) P2 as obtained by SPME-GC/MS.

Figure 4 shows typical chromatograms of pesto (Figure 4a for P1 and Figure 4b for P2, respectively). Overall, more than 70 peaks were identified, mostly related with basil volatile composition. Slight differences occurring in the volatile profile of P1 and P2 result from the differences in formulation (as reported in the Materials and Methods section). Among the major peaks, linalool, estragole (or methyl chavicol, only in P1), eugenol, eucalyptol, *trans*-α-bergamotene, *trans*-β-ocimene, β-myrcene arise from basil [28,29], as the major ingredient in pesto formulation, representing about 72–83% of the overall chromatographic area.

**Figure 5:**
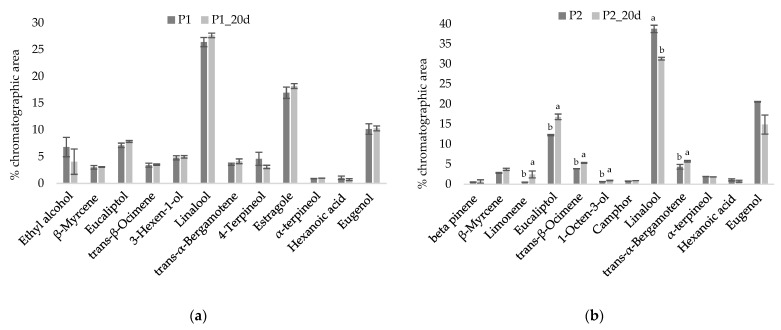
Comparison of the main volatile components (relative abundance) of (**a**) P1 and (**b**) P2 analyzed at the time of first opening and after 20 days from first opening (under S2). Different letters for each compound indicate significant differences (*p* < 0.05). If letters are not provided, no significant difference was observed.

The most representative volatile compounds, contributing about 86–90% of total chromatographic area, were selected for comparison between the reference (just-opened) sample and aged (20 d from first opening) sample for P1 (Figure 5a) and P2 (Figure 5b). As it can be inferred, the relative abundance of dominant compounds for aged samples basically reflects the profile of just-opened samples, thus confirming the substantial stability assessed through the sensory evaluation. For P1, the relative abundance of the key volatile compounds determined at the time of opening and after 20 d did not show significant difference (*p* < 0.05). For P2, instead, some significant differences (*p* < 0.05) were highlighted by comparison of the relative abundances at the time of opening and after 20 d, such as for linalool, which decreased with ageing, and for eucalyptol, limonene, and *trans*-β-ocimene which, instead, increased.

## 4. Conclusions

This study sought to investigate possible changes occurring in industrial pesto sauce after package opening, by using a mixed approach consisting of a simulation of household conditions and a rigorous scientific investigation of microbiological, sensory, and chemical parameters. This work could offer a first reference methodology for the SSL assessment of a wide range of shelf-stable products.

Results demonstrate that industrial shelf-stable pesto, irrespective of the intensity of domestic use, can be still suitable for consumption after 20 d from the first opening, upon refrigerated household storage. Therefore, our findings suggest the possibility to extend the indication of SSL for the studied pesto sauces from 5 to 20 d. Since the chosen samples well represent the array of industrial pesto sauces commercially available, based on the comparison of pH and a_w_, results could apply to the category of shelf-stable pesto sauces, having similar intrinsic parameters.

In a wider context, this work could have relevant practical outcomes both for the industry and the end consumer. The consciousness of the suitability for consumption even after the end of the indicated SSL could have consequences on the producer’s decision concerning the SSL to be declared on the food label. Indeed, selling a product with an increased SSL means paying attention to food waste, giving the image of a sustainable business. Furthermore, the extension of the labelled SSL based on its objective assessment would contribute to add value to the packaged food product, without anyway modifying ingredients, formulation, or production process. This innovation might increase competitiveness, leading the consumer to choose the product which lasts longer, rather than other products of the same commercial category but with a lower duration after opening. Overall, a company might improve its market positioning through the reassessment of the SSL of its products.

In addition, the increase of the useful period for food consumption after first opening would bring advantages for the end user, leading to an improvement of the household food management, with consequent cost savings. The enhanced consumer awareness following the modification of SSL in the label might lead to lower food wastes generation.

In conclusion, through the objective assessment of SSL and its effective communication to the end user, this study could have practical potential on domestic food waste reduction and on the overall sustainability of food chains.

## Figures and Tables

**Table 1:** Mean nutritional values of the used pesto brands (g per 100 g).

Constituents	Pesto 1 (P1)	Pesto 2 (P2)
Fat	46	48
Carbohydrate	9.8	3.2
Fibre	5.0	1.5
Protein	4.7	6.5
Salt	3.25	2.4

**Table 2:** Scenarios simulating consumer’s utilization of pesto sauce.

	Duration of Opening (min)	Removed Amount	Time (min) at T_amb_ after Closing	Repetitions
Scenario 1	2	2 tablespoons	0	1
Scenario 2	3	2 tablespoons	30	3

**Table 3:** Maximum observed counts for total aerobic mesophilic bacteria (TMB) and Clostridia (Cl) amongst the five domestic environments, for each day of sampling, scenario, and pesto. S: Scenario (1, 2); P: Pesto brand (1, 2).

Time after Opening (d)	TMB (CFU/g × 10^1^)				Cl (CFU/g)			
	S1 P1	S2 P1	S1 P2	S2 P2	S1 P1	S2 P1	S1 P2	S2 P2
5	96.4 ± 16.6 ^a^	7.7 ± 7.0 ^b^	10.4 ± 1.9 ^b^	31.5 ± 1.3 ^a^	n.d.	5.0 ± 7.1	n.d.	n.d.
11	2.7 ± 1.3 ^b^	96.4 ± 3.8 ^a^	1.8 ± 0.0 ^b^	79.3 ± 14.0 ^a^	n.d.	20.0 ± 0.0	n.d.	n.d.
16	3.2 ± 1.9 ^ab^	13.5 ± 5.1 ^a^	4.1 ± 0.6 ^ab^	1.8 ± 0.0 ^b^	n.d.	55.0 ± 21.2 ^a^	n.d.	5.0 ± 7.1 ^b^
20	2.3 ± 1.9 ^b^	3.6 ± 0.0 ^b^	0.9 ± 0.0 ^b^	8.1 ± 0.0 ^a^	5.0 ± 7.1	n.d.	n.d.	n.d.

Values are given as mean ± SD (*n* = 2). Different letters in the same line indicate significant differences (*p* < 0.05). If letters are not provided, no significant difference was observed. n.d.: not detected.

**Table 4:** Instrumental colour parameters (L* and a*) at time of first opening (t_0_) and after 5, 11, 16 and 20 d after first opening (t_5,_ t_11_, t_16_ and t_20_) for both pesto sauces (P1 and P2) managed in 5 different home environments (A–E) under two scenarios (S1 and S2).

L*			**t_5_**	**t_11_**	**t_16_**	**t_20_**
P1	S1	A	43.44 ± 3.99	46.51 ± 0.40	44.28 ± 5.09	45.21 ± 4.69
t_0_		B	45.43 ± 1.47	46.14 ± 1.49	46.57 ± 1.16	45.91 ± 1.90
47.98 ± 0.62^a^		C	45.07 ± 1.69	48.04 ± 1.87	46.71 ± 0.98	45.79 ± 2.21
		D	43.79 ± 1.78 ^b^	45.85 ± 0.40 ^ab^	46.51 ± 0.58 ^ab^	44.17 ± 1.97 ^b^
		E	43.65 ± 3.91	45.51 ± 1.72	47.55 ± 0.62	45.03 ± 2.51
	S2	A	46.68 ± 3.47	44.04 ± 1.08	45.47 ± 2.50	45.41 ± 0.37
		B	47.31 ± 0.28	45.02 ± 2.78	46.54 ± 0.37	47.65 ± 2.61
		C	46.40 ± 1.57	46.69 ± 0.69	48.53 ± 0.44	48.09 ± 0.59
		D	46.70 ± 1.14 ^ab^	44.32 ± 0.67 ^b^	45.56 ± 1.39 ^ab^	45.96 ± 1.48 ^ab^
		E	45.25 ± 0.45 ^bc^	40.87 ± 0.86 ^d^	47.74 ± 0.59 ^ab^	43.80 ± 1.84 ^c^
P2	S1	A	47.29 ± 1.26 ^a^	45.48 ± 0.89 ^ab^	45.68 ± 0.69 ^ab^	44.04 ± 1.95 ^b^
t_0_		B	44.54 ± 1.23	45.73 ± 1.11	45.70 ± 0.90	44.02 ± 0.42
44.34 ± 0.59 ^ab^		C	46.04 ± 2.04	46.29 ± 0.58	46.42 ± 1.86	44.13 ± 1.37
		D	46.60 ± 1.01 ^a^	45.45 ± 1.4 ^ab^	43.45 ± 1.45 ^b^	44.42 ± 0.67 ^ab^
		E	45.23 ± 0.64	45.87 ± 0.74	43.28 ± 1.87	45.27 ± 1.50
	S2	A	44.92 ± 2.15	46.08 ± 1.87	45.43 ± 2.19	45.20 ± 2.14
		B	45.52 ± 2.54	44.95 ± 1.37	45.93 ± 1.07	45.71 ± 1.62
		C	46.13 ± 0.85	44.97 ± 3.31	46.33 ± 2.96	48.08 ± 0.62
		D	46.77 ± 2.78	45.70 ± 2.47	45.82 ± 0.87	46.40 ± 1.69
		E	45.24 ± 3.63	44.74 ± 2.83	44.73 ± 1.27	46.06 ± 0.76
a*			**t_5_**	**t_11_**	**t_16_**	**t_20_**
P1	S1	A	−4.16 ± 0.41	−3.79 ± 0.13	−4.09 ± 0.38	−3.46 ± 0.45
t_0_		B	−3.99 ± 0.20	−3.90 ± 0.22	−3.99 ± 0.36	−3.54 ± 0.11
−3.82 ± 0.12 ^ab^		C	−4.17 ± 0.18 ^b^	−3.98 ± 0.11 ^b^	−3.89 ± 0.40 ^ab^	−3.32 ± 0.20 ^a^
		D	−3.63 ± 0.16	−3.55 ± 0.37	−3.66 ± 0.35	−3.78 ± 0.25
		E	−3.97 ± 0.09	−3.84 ± 0.44	−3.81 ± 0.20	−3.90 ± 0.11
	S2	A	−4.14 ± 0.35	−3.81 ± 0.41	−3.23 ± 0.30	−4.03 ± 0.14
−3.82 ± 0.12 ^bc^		B	−4.00 ± 0.12 ^c^	−3.41 ± 0.10 ^ab^	−3.28 ± 0.32 ^a^	−3.83 ± 0.12 ^bc^
		C	−3.94 ± 0.35	−3.82 ± 0.21	−3.84 ± 0.29	−3.60 ± 0.01
−3.82 ± 0.12 ^b^		D	−3.57 ± 0.14 ^ab^	−3.50 ± 0.26 ^ab^	−3.17 ± 0.29 ^a^	−3.66 ± 0.24 ^ab^
		E	−3.52 ± 0.31	−4.26 ± 0.23	−3.34 ± 0.29	−3.55 ± 0.63
P2	S1	A	−3.85 ± 0.27^b^	−3.73 ± 0.24 ^b^	−3.69 ± 0.07 ^b^	−3.50 ± 0.42 ^ab^
t_0_		B	−3.53 ± 0.20^ab^	−3.62 ± 0.44 ^ab^	−3.84 ± 0.20 ^b^	−3.36 ± 0.20 ^ab^
−3.0 ± 0.13^a^		C	−3.65 ± 0.40^ab^	−3.54 ± 0.14 ^ab^	−4.01 ± 0.14 ^b^	−3.57 ± 0.37 ^ab^
		D	−3.86 ± 0.22 ^b^	−3.67 ± 0.10 ^b^	−3.61 ± 0.32 ^b^	−3.58 ± 0.12 ^b^
		E	−3.45 ± 0.32	−3.59 ± 0.16	−3.47 ± 0.22	−3.42 ± 0.57
	S2	A	−3.54 ± 0.24 ^b^	−3.63 ± 0.13 ^b^	−3.27 ± 0.18 ^ab^	−3.33 ± 0.10 ^ab^
		B	−3.68 ± 0.46	−3.33 ± 0.18	−3.38 ± 0.17	−3.59 ± 0.34
		C	−3.75 ± 0.11 ^bc^	−3.50 ± 0.17 ^b^	−3.40 ± 0.23 ^b^	−3.97 ± 0.15 ^c^
		D	−3.63 ± 0.23 ^ab^	−3.89 ± 0.30 ^b^	−3.65 ± 0.27 ^ab^	−3.46 ± 0.33 ^ab^
		E	−3.90 ± 0.18	−3.14 ± 0.54	−3.36 ± 0.36	−3.40 ± 0.33

The t_0_ values were repeated only for samples having significance letters different than displayed. Values are given as mean ± SD (*n* = 3). Different letters in the same line indicate significant differences (*p* < 0.05). If letters are not provided, no significant difference was observed.

**Table 5:** a_w_ and pH values of ten types of commercial shelf-stable pesto sauces. Values are given as mean ± SD (*n* = 3). Different letters within each column indicate significant differences (*p* < 0.05). P1 and P2 were used in this study for the secondary shelf life assessment.

Pesto Brand (Code)	pH	a_w_	Indication of SSL (d)
P1	5.64 ± 0.07 ^a^	0.9046 ± 0.0004 ^g^	5
P2	4.18 ± 0.05 ^f^	0.9554 ± 0.0010 ^b^	5
P3	4.00 ± 0.01 ^g^	0.9579 ± 0.0006 ^b^	7
P4	4.51 ± 0.03 ^e^	0.9358 ± 0.0008 ^d^	14
P5	4.47 ± 0.04 ^e^	0.9239 ± 0.0006 ^e^	few days
P6	5.45 ± 0.02 ^b^	0.9574 ± 0.0012 ^b^	3–4
P7	4.72 ± 0.04 ^d^	0.9207 ± 0.0015 ^f^	5
P8	4.43 ± 0.02 ^e^	0.9508 ± 0.0008 ^c^	4
P9	4.44 ± 0.02 ^e^	0.9667 ± 0.0013 ^a^	3
P10	4.91 ± 0.02 ^c^	0.8447 ± 0.0008 ^h^	10

## Supplementary Materials

The following are available online at https://www.mdpi.com/article/10.3390/foods10081948/s1, Figure S1: Samples of pesto sauce P2 prepared for the sensory analysis. Each of the five triplets consisted of two identical samples and one different sample, which was randomly selected between the stored pesto (11 days after opening, second scenario) and the control one, Figure S2: Pictures of the commercial pesto sauces (a) P1 and (b) P2, at the time of opening (t_0_) and after 5, 11, 16 and 20 days of household storage.
