# Supplementary material for: Domestic Use Simulation and Secondary Shelf Life Assessment of Industrial Pesto alla genovese
Source: Foods. 2021 Aug 21;10(8):1948. doi: 10.3390/foods10081948 (PMC8391206; doi:10.3390/foods10081948)
Supplement: Supplementary file 1 [file foods-10-01948-s001.zip › foods-1340228-supplementary.pdf]

Supplementary Material

Domestic use simulation and secondary shelf life assessment of industrial *Pesto alla genovese*

Carola Nicosia [1], Patrizia Fava [1,2], Andrea Pulvirenti [1,2], Andrea Antonelli [1,2], Fabio Licciardello [1,2,*]

[1] Department of Life Sciences, University of Modena and Reggio Emilia, 42122 Reggio Emilia, Italy; carola.nicosia@unimore.it (C.N.); patrizia.fava@unimore.it (P.F.); andrea.pulvirenti@unimore.it (A.P.); andrea.antonelli@unimore.it (A.A.)

[2] Interdepartmental Research Centre for the Improvement of Agro-Food Biological Resources (BIOGEST-SITEIA), University of Modena and Reggio Emilia, 42122 Reggio Emilia, Italy

* Correspondence: fabio.licciardello@unimore.it;

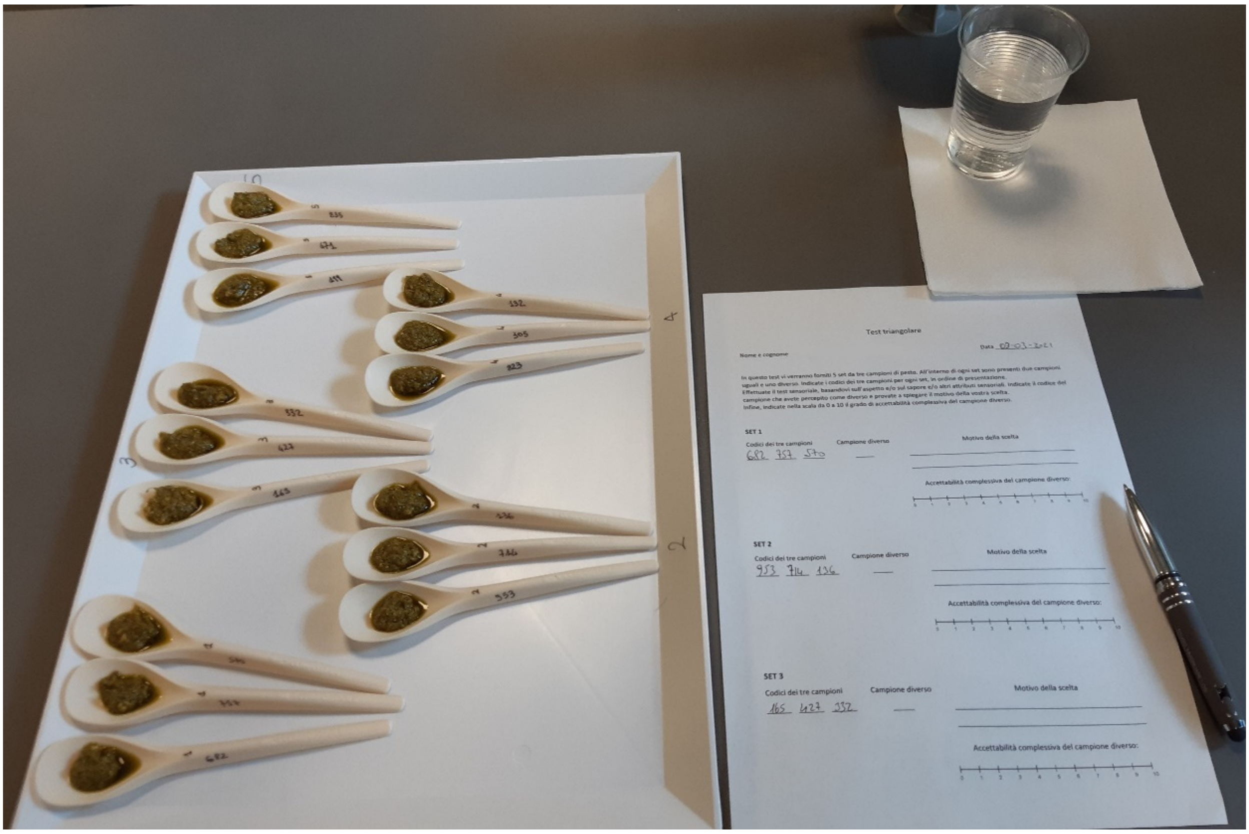

Figure S1. Samples of pesto sauce P2 prepared for the sensory analysis. Each of the five triplets consisted of two identical samples and one different sample, which was randomly selected between the stored pesto (11 days after opening, second scenario) and the control one.

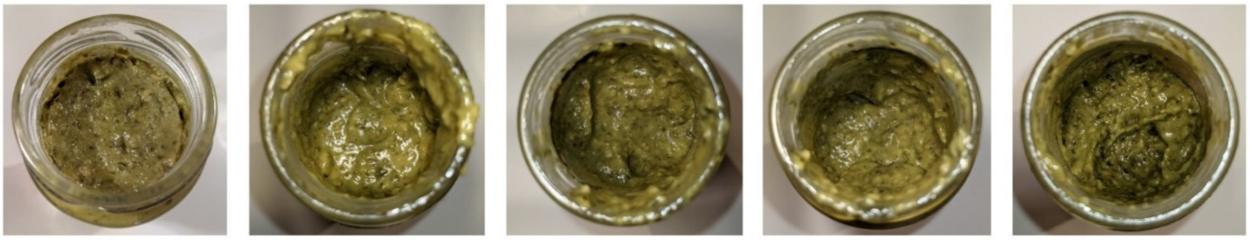

t_0 t_5 t_{11} t_{16} t_{20}

(**a**)

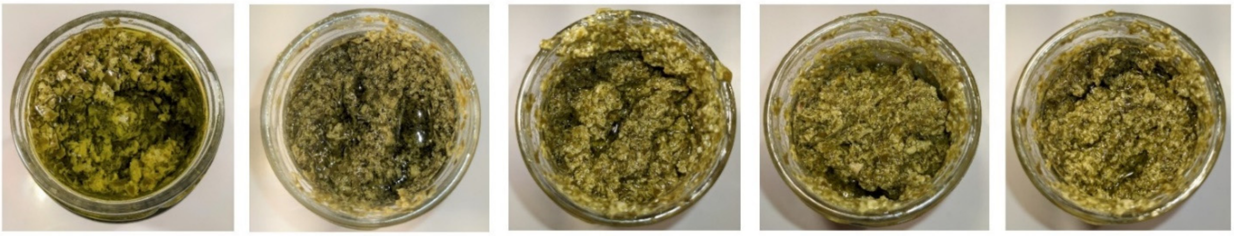

t_0 t_5 t_{11} t_{16} t_{20}

(**b**)

Figure S2. Pictures of the commercial pesto sauces (a) P1 and (b) P2, at the time of opening (t_0) and after 5, 11, 16 and 20 days of household storage.
